# Validation of Simultaneous Volumetric and HPLC Methods for the Determination of Pridinol Mesylate in Raw Material

**DOI:** 10.1155/2013/540676

**Published:** 2013-10-02

**Authors:** Laura D. Simionato, Leonardo Ferello, Sebastián Stamer, Patricia D. Zubata, Adriana I. Segall

**Affiliations:** Cátedra de Calidad de Medicamentos, Facultad de Farmacia y Bioquímica, Universidad de Buenos Aires, CONICET, Junín 956, 1113 Buenos Aires, Argentina

## Abstract

Simple, sensitive, and economical simultaneous volumetric and HPLC methods for the determination of pridinol mesylate in raw material have been developed. The volumetric method is based on the reaction of pridinol with sodium lauryl sulphate in diluted sulphuric acid. Dimethyl yellow was used as indicator to detect the end point of the titration in aqueous/organic layer. The HPLC method for the determination of pridinol mesylate employs a reverse phase C18 column at ambient temperature with a mobile phase consisting of acetonitrile: 0.05 M potassium dihydrogen phosphate, pH adjusted to 5.0 (1 : 2, v/v). The flow rate was 0.8 mL/min. Quantitation was achieved with UV detection at 258 nm based on peak area. Both methods were found to be suitable for the quality control of pridinol mesylate in raw material.

## 1. Introduction

**Figure 1:**
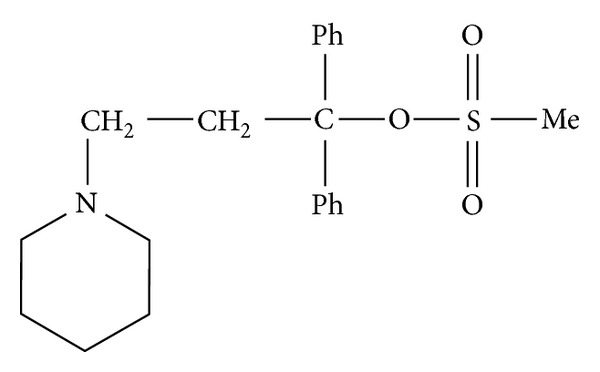
Pridinol mesylate.

Pridinol mesylate (Figure 1) is a CNS acting muscle relaxant used for treating muscle spasms [1]. It is administered orally and by intramuscular injection, and it has also been applied in topical preparations [1]. However, pridinol mesylate is most frequently found in associations with nonsteroidal anti-inflammatory agents, including sodium or potassium diclofenac and meloxicam. The usual dosis is 4.0 mg. 

A literature survey revealed some high-performance liquid chromatographic methods for the quantitation of pridinol mesylate in the presence of its relevant degradation impurities [2] or in the presence of its process-related impurities [3] in bulk drug. Both methods use a C18 column and mixtures of potassium phosphate buffer, methanol and 2-propanol as the mobile phase [2, 3]. Another analytical technique for pridinol mesylate described in the literature is based on the liquid chromatographic determination of this drug in pharmaceutical formulations with meloxicam [4].

The present paper describes a simple, rapid, precise, accurate, and economic volumetric method for the quantitation of pridinol mesylate in raw material. Results of precision and accuracy of the volumetric method were comparable with an isocratic reversed-phase HPLC method developed for the determination of pridinol mesylate in raw material. 

The method was validated by following the analytical performance parameters suggested by the International Conference on Harmonization (ICH) [5]. Previously we have described an HPLC method for the quantitation of Meprednisona in tablets, and we use the same lineaments [6].

## 2. Materials and Methods

Pridinol mesylate (99.6%) was obtained from Aldrich-Sigma (Saint Louis, MO, USA). Acetonitrile used was HPLC Grade, J. T. Baker, Estado de Mexico, Mexico. Potassium dihydrogen phosphate was from AR Grade, J. T. Baker, Estado de Mexico, Mexico; Sulphuric acid, AR Grade (Merck, Darmstadt, Germany); Dichloromethane, AR Grade (Mallinckrodt, New York, USA); Dimethyl yellow, AR Grade (Aldrich-Sigma, Saint Louis, MO, USA); and Sodium Lauryl Sulphate (Flamaquímica, Argentina). Papaverine hydrochloride (98.9%) was obtained from Drogueria Saporiti, Buenos Aires, Argentina. Distilled water was passed through a 0.45 *μ*m membrane filter.

### 2.1. Volumetry

#### 2.1.1. Reagent and Materials

10% (w/v) sulphuric acid was prepared by appropriate dilution of concentrated Sulphuric acid. Dimethyl yellow 1.0% (w/v) was prepared in dichloromethane. Sodium lauryl sulphate 0.01 N was prepared by diluting 2.88 g in 1000 mL of distilled water. 

#### 2.1.2. Standardization

Sodium lauryl sulphate solution 0.01 N was standardized with papaverine hydrochloride. 50 mg of papaverine hydrochloride accurately weighed was placed in a 100 mL conical flask and dissolved in 20 mL of distilled water and 5 mL of 10% (w/v) sulphuric acid. 20 mL of dichloromethane and 3 drops of indicator were then added. After being vigorously shaken, the solution was titrated with 0.01 N sodium lauryl sulphate until the organic layer changed from yellow to red-orange at the end of titration.

### 2.2. General Procedure

Each amount of pridinol mesylate was accurately weighed, transferred to a 100 mL conical flask, and dissolved in 20 mL of distilled water and 5 mL of 10% (w/v) sulphuric acid. 20 mL of dichloromethane and 3 drops of indicator were added afterwards. After vigorous shaking, the solution was titrated with 0.01 N sodium lauryl sulphate previously standardized. After each amount of titrant was added, the flask was shaken vigorously until the organic layer changed from yellow to red-orange at the end of titration.

### 2.3. Method Validation

#### 2.3.1. Linearity

Linearity solutions were prepared at five concentrations levels from 25% (w/v) to 125% (w/v) of analyte concentration. 

#### 2.3.2. Precision

Precision of the method was checked by carrying out six independent assays of pridinol mesylate raw material. Intermediate precision was performed by analyzing the samples by two different analysts on different days. 

#### 2.3.3. Accuracy

The accuracy was evaluated by the recovery studies at concentration levels of 75, 100, and 125% (w/v) (3 samples each). The amount of pridinol mesylate recovered in relation to the added amount was calculated.

### 2.4. HPLC Method

#### 2.4.1. Equipment

The HPLC system consisted of a dual piston reciprocating Spectra Physics pump (Irvine, CA, United States, Model ISO Chrom. LC pump), a UV-Vis Hewlett Packard detector (Model 1050), a Hewlett Packard integrator (Loveland, CO, United States, Series 3395), and a Rheodyne injector (Model 7125).

#### 2.4.2. Chromatographic Conditions

The analytical column was a reversed phase C18 column Keystone ODS/A (250 × 3 mm, 5 *μ*m) Keystone Scientific Inc. The separation was carried out under isocratic elution with acetonitrile: 0.05 M potassium dihydrogen phosphate, pH adjusted to 5.0 (1 : 2, v/v). The flow rate was 0.8 mL/min. The wavelength was monitored at 258 nm, and the injection volume was 20 *μ*L. The HPLC was operated at ambient temperature. Under these conditions, the retention time (*t*_*R*_) of pridinol mesylate was approximately 4.8 min. 

#### 2.4.3. Standard Solutions

A standard stock solution of pridinol mesylate was prepared at a concentration of 0.88 mg/mL in mobile phase and filtered through a 0.2 *μ*m nylon membrane (25 mm disposable filter; Cat. N° Y02025WPH *μ*icroclar, Buenos Aires, Argentina). The solution remained stable for 3 days stored at 2–8°C, in the dark. 

#### 2.4.4. Sample Preparation

Approximately 25 mg of pridinol mesylate raw material was accurately weighted and diluted in mobile phase in order to obtain a concentration of 0.88 mg/mL. The sample was filtered through a 0.2 *μ*m nylon membrane (25 mm disposable filter; Cat. N° Y02025WPH *μ*icroclar, Buenos Aires, Argentina).

### 2.5. Method Validation

#### 2.5.1. System Suitability

Relative standard deviations (RSD) values of the peak area, tailing factor, retention time, capacity, and theoretical plates were the chromatographic parameters selected for the system suitability test [7].

#### 2.5.2. Specificity

Forced degradation studies were performed to evaluate the specificity of the method. Degraded samples were prepared by refluxing 1.76 mg/mL pridinol mesylate working standard with acid (6 N hydrochloric acid), base (5 N NaOH), water, and 30% hydrogen peroxide and refluxing for at least 30 min. The drug was subjected to thermal degradation in the solid state in an open container in an oven at 50°C for 1.5 h and to photochemical degradation (a solution was transferred to a container and exposed to daylight for 96 h). After each degradation treatment, samples were allowed to cool at room temperature and diluted, to the same concentration as that of the standard solution, after being neutralized. After degradation, samples were analyzed using the methodology and the chromatographic conditions described.

#### 2.5.3. Linearity

Linearity solutions were prepared at nine concentrations levels from 2.5% (w/v) to 150% (w/v) of analyte concentration. 

#### 2.5.4. Precision and Accuracy

Both reproducibility and accuracy studies were evaluated by carrying out nine independent assays at concentration levels of 80, 100, and 120% (w/v) (3 samples each). Raw material was used in these assays. The amount of pridinol mesylate recovered was calculated. 

#### 2.5.5. Robustness

 Robustness was performed by deliberately changing the chromatographic conditions. The relative organic portion ratio of the eluent was varied by 0.8 to 2.0, while pH was varied by ±1.4 units. RSD, retention time, tailing, and theoretical plates were evaluated. 

## 3. Results and Discussion

In acidic solutions, the nitrogenous drugs are present in their positively charged form. The improved dissolution of pridinol mesylate at lower pH is probably due to the protonation of the piperidinic nitrogen (pKa = 9.7) [8]. The anionic titrant interacts with the positively charged pridinol to form ion-pair or ion-associate complexes which are extractable into organic solvents. The aqueous to organic solvent ratio 1 : 1 was the more suitable for the extraction of ion pair or ion-associate complexes. At the end point, the titrant interacts with the basic dye to form ion-pair or ion-associate complexes which changes the color of the organic layer from yellow to red-orange. 

The proposed procedure has been satisfactorily applied to the quantitation of pridinol mesylate in raw material.

The linearity of the volumetric method was determined by analysis of three replicates of five concentrations of standard solutions from 25% (w/v) to 125% (w/v). The calibration curve showed good linearity over the concentration range. The correlation coefficient (“*r*”) value was 0.9998. Typically, the regression equation for the calibration curve was found to be *y* = 0.2605061*x* + 0.0254146. The linearity of the calibration graphs was validated by the high value of the correlation coefficient and the intercept value that was not statistically (*P* = 0.05) different from zero (Table 1). 

The precision of an analytical procedure expresses the closeness of the agreement between a series of measurements obtained from multiple sampling of the same homogeneous sample under the prescribed conditions.

The intraday precision of the volumetric method was performed by assaying the samples on two different days by two different analysts. The results were given both individually and as the average. For each precision assay the results were as follows: mean values 100.1 and 100.0% and RSD 1.1% and 1.0%. *T* test comparing two samples with 95% confidence for 10 degrees of freedom disclosed that both results were not significantly different *inter se*  (*t*_*n*−2,*α*:0.05_) = 2.23 (Table 2).

Method accuracy of the volumetric method was also demonstrated by plotting the amount of pridinol mesylate measured against the amount present in the samples, both expressed in mg. Linear regression analysis rendered slopes not significantly different from 1 (*t*-test *P* = 0.05), intercepts not significantly different from zero (*t*-test *P* = 0.05), and *r* = 0.9999; the RSD was 0.2. The experimental *t* of the recovery percentage whose value was 0.750 was also studied, being it far below the 2.306 established in the tabulated *t* (95% level of probability, 8 d.f.) (Table 3). 

The described reverse-phase liquid chromatography method was developed to provide a rapid quality control determination of pridinol mesylate in raw material. Validation of the method was performed according to ICH. This method uses a simple mobile phase. All samples were analyzed using the assay chromatographic conditions described. 

The analytical column was equilibrated with the eluting solvent system used. After an acceptably stable baseline was achieved, the standards and then the samples were analyzed. 

System suitability results were calculated according to the USP 32 〈621〉 from typical chromatograms. Instrument precision was determined by six successive injections of the standard preparation provided a relative standard deviation (RSD) below 1.5%. Peak asymmetry or tailing factor, *T*, was calculated as *T* = *W*_0.05_/2*f*, where *W*_0.05_ is the distance from the leading edge to the tailing edge of the peak, measured at 5% of the peak height from the baseline, and *f* is the distance from the peak maximum to the leading edge of the peak. The tailing factor did not exceed 1.5. The RSD of peak area response and retention time showed the satisfactory repeatability of the system (<1.5%) (Table 4). 

Stability of the standard solution and sample preparation was studied by injecting the prepared solution at periodic intervals into the chromatographic system up to about 72 hours stored at 2–8°C. 

Degradation was indicated in the stressed sample by a decrease in the expected concentration of the drug and increased levels of degradation products. Pridinol mesylate was degraded to different products under acid, base, and oxidation (Table 5). In addition, there was no interference regarding the retention time of pridinol mesylate and its degradation products. 

The linearity of the HPLC method was determined by analysis of three replicates of nine concentrations of standard solutions (ranging from 0.44 to 24.70 *μ*g injected). The calibration curve showed good linearity over the concentration range. The correlation coefficient (“*r*”) value was 0.9995. Typically, the regression equation for the calibration curve was found to be *y* = 251840*x* + 70531. The linearity of the calibration graphs was validated by the high value of the correlation coefficient and the intercept value that was not statistically (*P* = 0.05) different from zero (Table 6). 

The system precision is a measure of the method variability that can be expected for a given analyst performing the analysis and was determined by performing six replicate analyses of the same working solution. The relative standard deviation (RSD) obtained was 0.4.

The precision is usually expressed as the RSD of a series of measurements. The reproducibility and accuracy studies were evaluated by recovery studies with 9 samples of one commercial formulation studied (*n* = 3 for 80%, 100%, and 120%) which indicated that the mean recovery was 100.2% and the RSD was 1.0.

Method accuracy was also demonstrated by plotting the amount of pridinol mesylate found against the amount present in the sample, both expressed in mg. Linear regression analysis rendered slopes not significantly different from 1 (*t*-test *P* = 0.05), intercepts not significantly different from zero (*t*-test *P* = 0.05), and *r* = 0.9995. The experimental *t* of the recovery percentage was also studied, showing a value of 0.750, far below the 2.306 established in the tabulated *t* (95% level of probability, 8 d.f.) (Table 7). 

The robustness of an analytical procedure is a measure of its capacity to remain unaffected by small, but deliberate variations in method parameters and provides an indication of its reliability during normal usage.

Robustness of the method was investigated under a variety of conditions including changes of pH and percentage of acetonitrile in the mobile phase. 

The effect on retention time, theoretical plates and tailing factor can be seen in Table 8. An increase in acetonitrile proportion reduces both retention time and theoretical plates. It was found that retention time of pridinol mesylate was significantly affected by pH changes. 

The volumetric method proposed is simple, rapid, and inexpensive and can therefore be applied to the determination of pridinol mesylate in raw material. Method validation yielded good results and included precision and accuracy. 

A straightforward, specific, linear, precise, and accurate RP-HPLC method has been developed and validated for quantitative determination of pridinol mesylate in raw material. The method is very simple and specific, as the peak is well separated from its impurities with total runtime of 15 min, which makes it especially suitable for routine quality control analysis work. 

## Figures and Tables

**Table 1:** Volumetric linearity.

% (w/v) of nominal value	Weighed (*μ*g)	Volume consumed
25	0.0218	5.7
50	0.0370	9.6
75	0.0545	14.2
100	0.0710	18.5
125	0.0869	22.7

Slope^a^	0.2605061 ± 1.4117187	
Intercept^b^	0.0254146 ± 83.5200138	

^a^Confidence limits of the slope (*P* = 0.05).

^b^Confidence limits of the intercept (*P* = 0.05).

**Table 2:** Volumetric precision.

Sample no. of analyst 1	Weighed (mg)	Percentage	Sample no. of analyst 2	Weighed (mg)	Percentage
1	71.0	100.0	1	69.8	100.0
2	70.9	100.1	2	70.2	100.0
3	72.4	100.1	3	71.1	99.8
4	71.6	100.2	4	71.0	100.0
5	72.8	100.1	5	69.9	99.9
6	71.1	100.3	6	71.6	100.2

Mean	71.6	100.1		70.6	100.0
RSD	1.1	0.1		1.0	0.1

**Table 3:** Volumetric accuracy.

% of nominal value	Added amount (mg)	Found amount (mg)	Recovery (%)	Average recovery (*n* = 3)	RSD (%)
75	54.1	54.0	100.0	100.2	0.3
	54.0	54.0	100.0		
	55.3	55.6	100.5		

100	71.0	70.9	99.8	99.9	0.1
	71.7	71.6	99.9		
	70.2	70.1	99.9		

125	87.0	87.0	100.0	99.9	0.1
	86.0	85.8	99.8		
	87.8	87.7	99.9		

Mean (*n* = 9)				100.0	0.2

**Table 4:** System suitability.

Parameter	Minimum value	Maximum value	Average	RSD (%)
Retention time	4.869	4.880	4.871	0.29
Area	4674893	4745226	4710059.5	1.05
Capacity	2.13	2.14	2.135	0.33
Tailing factor	1.0	1.04	1.02	2.77
Theoretical plates	8636.6	9502.35	9069.47	6.77

**Table 5:** Selectivity.

Condition	Time (h)	% of pridinol mesylate	RRT of degradation products
Acid (6 N HCl, reflux)	0.5	9.8	0.29, 0.33, 0.4, 0.49, 1.24, 1.88, 2.26
Base (5 N NaOH, reflux)	0.5	11.5	0.23, 0.33
Hydrogen peroxide 100 vol (reflux)	0.5	68.2	0.33, 1.16, 1.6
Dry heat, 50°C (solid)	1.5	99.5	0.35
Daylight exposure	96	100.4	Non detected

RRT: relative retention time.

**Table 6:** HPLC linearity.

% of nominal value	Injected (*μ*g)	Average peak area response	RSD
2.5	0.44	124724.7	0.6
5	0.88	240208.5	1.3
10	1.76	465203.0	0.2
25	4.40	1253760.0	0.3
50	8.80	2375012.0	1.4
75	12.36	3192030.0	1.2
100	17.60	4618572.7	0.1
125	21.60	5467281.0	0.5
150	24.70	6203272.0	0.8

Slope^a^	251840.13 ± 812125.44		
Intercept^b^	70530.92 ± 11502975.25		

^a^Confidence limits of the slope (*P* = 0.05).

^b^Confidence limits of the intercept (*P* = 0.05).

**Table 7:** HPLC precision and accuracy.

% of nominal value	Added amount (mg)	Found amount (mg)	Recovery (%)	Average recovery (*n* = 3)	RSD (%)
80	33.0	32.8	99.3	99.4	0.4
	32.0	31.6	99.0		
	32.6	32.1	99.8		

100	21.0	20.9	99.5	100.3	0.7
	20.7	20.9	100.9		
	21.3	21.4	100.5		

120	25.2	25.3	100.4	100.8	0.4
	24.7	25.0	101.2		
	25.0	25.2	100.8		

Mean (*n* = 9)				100.2	1.0

**Table 8:** Robustness.

Mobile phase	RT pridinol mesylate	RSD	Tailing	*N*	%	RSD
Acetonitrile : buffer (0.8 : 2)	8.747	0.11	1.02	30276	101.9	0.21
Acetonitrile : buffer (2 : 2)	2.296	0.03	1.07	8438.2	99.7	1.70
Acetonitrile : buffer (2 : 1) pH 5	5.790	0.23	1.03	13370.3	101.7	0.48
Acetonitrile : buffer (2 : 1) pH 6.4	6.588	0.24	1.00	69484.9	99.3	0.44
